# Neuromuscular and occlusion analysis to evaluate the efficacy of three splints on patients with bruxism

**DOI:** 10.1186/s12903-023-03044-5

**Published:** 2023-05-25

**Authors:** Qun Lei, Dong Lin, Yuyu Liu, Kaijin Lin, Wenxiu Huang, Dong Wu

**Affiliations:** 1 Fujian Key Laboratory of Oral Diseases & Fujian Provincial Engineering Research Center of Oral Biomaterial & Stomatological Key Laboratory of Fujian College and University, School and Hospital of Stomatology, Fujian Medical University, Fuzhou, China; 2 Fujian Medical University, Fuzhou, China

**Keywords:** Bruxism, Splint, Electromyography, Occlusion, Transcutaneous electrical nerve stimulation

## Abstract

**Objective:**

Occlusal splints are always applied on individuals with bruxism to reduce tooth wear and relieve orofacial symptoms such as myofascial pain. The stomatognathic system is mainly composed of tooth, occlusion, masticatory muscles, and temporomandibular joint. The occlusion and masticatory muscles function are regarded as the important parameters for evaluating the stomatognathic system state objectively. However, the effects of occlusal splints on individuals with bruxism is rarely elucidated from accurate neuromuscular analysis and occlusion evaluation. The aim of the present study was to estimate the effects of three different splints (two clinically common full coverage occlusal splint and an modified anterior splint) on subjects with bruxism using K7-J5 neuromuscular analysis system and Dental Prescale II (DP2) to evaluate occlusion.

**Methods:**

Sixteen subjects claimed to be suffering from nocturnal bruxism,with complete dentition and stable occlusal relationship, were selected for study.The intermaxillary space and the baselines of EMG-activity of the anterior temporalis and masseter were recorded for all the subjects. The participants was treated with three different splints, and outcomes were estimated by comfort index, occlusion and surface electromyography of anterior temporalis and masseter.

**Results:**

At teeth clenched position, EMG data were significantly lower in the participants with use of modified anterior splint than with hard, soft occlusal splint or without splint (*p* < 0.05). The maximum bite force and bite area occur in subjects without use of splint, while the minimal occur in subjects with use of modified anterior splint. Intermaxillary space increased and masticatory muscles presented significant reduction of EMG data at rest position as a result of J5 (*p* < 0.05).

**Conclusion:**

Modified anterior splint seems to be more comfortable and effective in reducing occlusion force and electromyographic activity of anterior temporalis and masseter for subjects with bruxism.

## Background

Bruxism is a frequently-occurring and common bad habit in stomatology. Compared with the bite force in physiological state, it is greater significantly, lasts for a longer time and has no rhythm in bruxism patients during grinding, which will bring obvious damage to the dental hard tissue, periodontal tissue, implant, temporomandibular joint, neuromuscular tissue, etc. The bruxism occurring in the waking state during the day is called “day bruxism”, while the bruxism occurring in the unconscious state of sleep at night is called “night bruxism”. Patients with bruxism is almost accompanied by greater muscular tension and obvious tooth wear [1–3], especially in patients with night bruxism, and those who sleep with them can often hear squeaky tooth grinding. Bruxism was originally thought to be a central nervous system problem, associated with anxiety, tension, and the release of stressful [4]. Recent studies have shown that bruxism may be caused by a synergistic effect of multiple factors, such as genetic factors [5], neuropsychological factors (long-term anxiety and tension) [6, 7], physiological and pathological factors (malocclusion, snoring, sleep disorder) [8–11], and bad lifestyle habits (smoking, alcoholism, excessive caffeine intake) [12–15].

Bruxism is harmful to body. For teeth, long-term grinding or clenching habit will cause dentofacial morphology of tooth hard tissue change, such as attrition, defect, and forming thin wall and weak cusps, make tooth sensitive and even get pulpitis. Thin wall and weak cusps formed can then cause occlusal trauma. Occlusal trauma and prolonged abnormal biting pressures will lead to crown or root fractures, accelerate periodontal breakdown, such as gingival atrophy, alveolar bone absorption, resulting in tooth mobility and displacement [16–18]. Severe dental attrition can lead to orofacial dysfunction, such as decreased vertical distance, temporomandibular joint disorders, disc perforation, orofacial muscular pain, and even restricted mouth opening [19–22]. Therefore, for patients with bruxism, we need to take some measures to protect oral and maxillofacial system.

The splint is one of the most commonly used removable device for treating oral and maxillofacial system diseases. It can change the bite touch state between upper and lower teeth, adjust the position relationship between the upper and lower jaw, improve temporomandibular joint and masticatory muscle function. The occlusion and masticatory muscles function are regarded as the important parameters for evaluating the stomatognathic system state objectively However, the effects of occlusal splints on individuals with bruxism is rarely elucidated from accurate neuromuscular analysis and occlusion evaluation.

There are several types of splints. Reference the range of the dentition covered, it can be divided into the part and full coverage occlusal splint;according to materials, it can be divided into hard and soft occlusal splint; based on the therapeutic effect, it can be divided into stabilization splint and repositioning splint. In this study, two clinically common full coverage occlusal splint and an modified anterior splint were selected to be used by participants with bruxism for evaluating the clinical effects through subject feeling, accurate neuromuscular analysis and occlusion evaluation.

## Methods

### Patient selection

This study was approved by the Biomedical Research Ethics Committee of Stomatology Hospital of Fujian Medical University, China. The informed consents were obtained from all participation in the study prior to understanding the aim of this investigation. The exclusion criteria were as follows: (1) presence of neuromuscular disease, (2) presence of mental disorders; (3) presence of heart disease (3) presence of temporomandibular joint disorders (clicking sounds in opening and closing, mouth-opening limited, locking or luxation of the joint, pain in temporomandibular joints). Sixteen subjects with complete dentition, stable occlusal relationship, and age ranging from 20 to 45 years old, were selected to participant in this study. They claimed to be suffering from nocturnal bruxism for at least six months, the voice of clenching and grinding teeth may be loud enough to bother the person or sleep partner. During the time of the study, other dental treatment was not proceed.

### Occlusal splint design

**Fig. 1:**
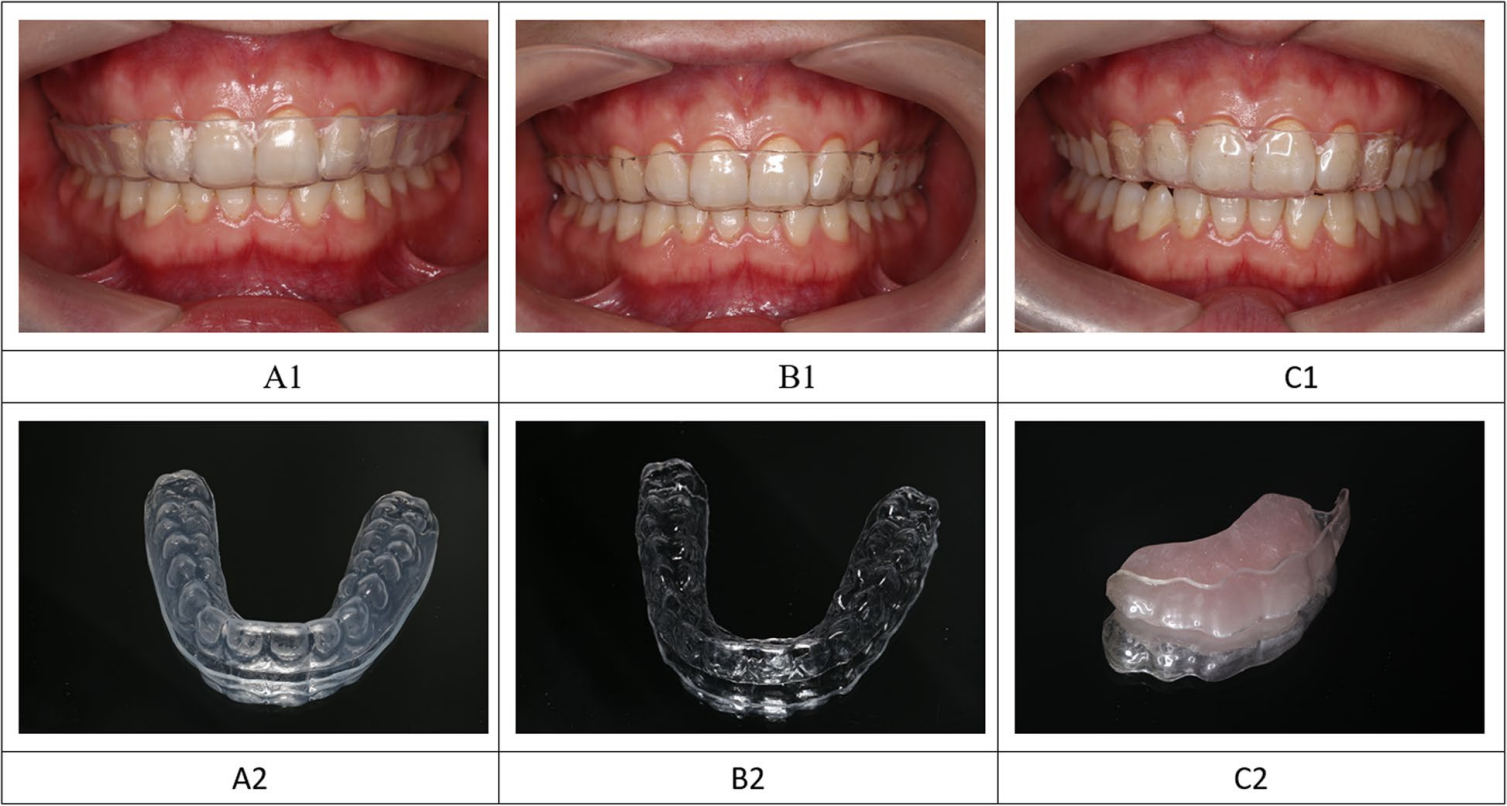
Three different splints. A1, A2: soft occlusal splint; B1, B2: hard occlusal splint; C1, C2: modified anterior splint

Models for participant were prepared by using alginate and hard plaster. A removable personalized well-fitting hard and soft occlusal splint were fabricated for the entire maxillary dentition of each subjects. a 1.5 mm-thick thermoplastic hard occlusal splint, and 1.5 mm-thick soft thermoplastic occlusal splint in upper model were fabricated by vacuum pressure molding device with an infrared heater. The modified anterior splint consists of a 1.5 mm-thick thermoplastic partial hard occlusal splint covering the anterior maxillary teeth and palatine-side flat bite plate made of self-curing resin, which only contacted with the lower incisor while biting. The occlusal thickness of the flat bite plate is decided according to free-way space (Fig. 1).

### Comfort index (Numerical Rating Scale)

Numerical Rating Scale was used to assessed patient comfort index on a scale from 0 to 10 (rating scale: 0, no discomfort; 1–3, mild discomfort; 4–6, moderate discomfort; 7–10,severe discomfort). Patient were asked to record the NRS for every splint after putting on three different splints in the maxilla and exercise the occlusal function for 10 min.

### Electromyographic detection

Surface electromyography (EMG) recordings were performed using the Cranio-Mandibular K7 evaluation system (Myotronics-Noromed, Inc., Tukwila, WA, USA) with eight-channel high quality silver-silver chloride surface electrodes. After cleaned with alcohol and dry, self-adhesive EMG electrodes were applied to the center of the bilateral anterior temporal muscles and masseter with the tip of each electrode parallel to the muscle fibers. Participants were asked to sit on a chair with their back upright, uncrossed arms and legs, and natural head position in a relaxed state during recording EMG [23]. Measurements and data analyses were performed by a single examiner. Fifteen-second data of the EMG recordings expressed in μV were recorded during each of the following situations: (1) without splint at rest position before J5 Myomonitor transcutaneous electrical nerve stimulation treatment: participants were not installed on splint with mandible at rest and teeth separated before J5 Myomonitor transcutaneous electrical nerve stimulation treatment; (2) without splint at maximum intercuspation position: subjects clenched their teeth at maximum intercuspation position without splint; (3) with soft occlusal splint at the maximum intercuspation position: participants were installed on soft occlusal splint with teeth clenched at maximum intercuspation position; (4) with hard occlusal splint at maximum intercuspation position: participants were installed on hard occlusal splint with teeth clenched at maximum intercuspation position; (5) with modified anterior splint at maximum intercuspation position: participants were installed on modified anterior splint with teeth clenched at maximum intercuspation position; (6) without splint at rest position after J5 Myomonitor transcutaneous electrical nerve stimulation treatment: participants were not installed on splint with mandible at rest and teeth separated after J5 Myomonitor transcutaneous electrical nerve stimulation treatment.

### Occlusal force evaluation with Dental Prescale II (DP2)

The bite forces at the maximum intercuspation position were estimated by accurate quantifiable computerized occlusal analysis systems Dental Prescale II (DP2, GC Corp., Tokyo, Japan), which were used to compare maximum occlusal force change before and after three different occlusal splints applied to subjects. After familiar with the measurement procedure and instructions, the sensor was inserted in the participants mouths aligned centrally with the midline of the upper incisor and then they were instructed to bite with maximum biting force at maximum intercuspation. In bite force estimation by Dental Prescale II, they were asked to bite on sensor the pressure-sensitive film to reach maximally clench for about 3 s, then the films were scaned and analysed by a color image scanner analyzer (Bite Force Analyzer; GC, Tokyo, Japan), which was used to estimate the bite force (N) and bite area (mm^2^) before and after use of occlusal splints. There was an interval for relaxation between each recording. The next measurement was conducted after muscle strength returns to normal and subjects self-reported that there was no discomfort or fatigue.

### Intermaxillary space before and after J5 Myomonitor transcutaneous electrical nerve stimulation (TENS)

Before the J5 Myomonitor TENS application, the intermaxillary space, baseline EMG activity at rest position, EMG activity with three different splints or without splints at teeth clenched position were recorded for all the subjects. For record the intermaxillary space, one point on the tip of the nose and the chin were marked, the distance between two points at rest position and maximum intercuspation position were measured using a caliper. The difference between the two data was regarded as the intermaxillary space before TENS. After intermaxillary space, baseline EMG activity, and EMG activity with three different splints were recorded, J5 Myomonitor TENS Unit device were applied (Myotronics-Noromed, Inc., Tukwila, WA, USA). According to the instructions, two active electrodes (anodes) from channel A were placed anterior to the tragus on both sides, and the third electrode (cathode) on the midline of the neck, below the hairline, while two active electrodes (anodes) from channel B were positioned on trapezius, and the third electrode (cathode) on the neck right below the previous electrode from channel A.40 min muscle relaxation from TENS were performed, then the intermaxillary space and EMG activity were remeasured again and recorded as the data after TENS.

### Statistical methods

The data were expressed as Mean ± SD. Statistical analyses were performed using software program (IBM SPSS Statistics, v25.0; IBM Corp). One-way analysis of variance (ANOVA) and Friedman M comparisons were used to compare participants without splints and with three different splints. A *p*-value of < 0.05 was considered statistically significant.

## Results

### Comfort index (Numerical Rating Scale)

In terms of comfort, subjects with use of hard occlusal splint reported worse discomfort compared with subjects with use of soft occlusal splint and modified anterior splint (hard occlusal splint vs soft occlusal splint: 3.38 ± 1.02 vs 1.63 ± 0.72], *P* < 0.05; hard occlusal splint vs modified anterior splint: 3.38 ± 1.02 vs 2 ± 0.73, *P* < 0.05). No statistically significant differences with use of soft occlusal splint were found compared with use of modified anterior splint (1.63 ± 0.72vs 2 ± 0.73, *P* > 0.05).

### Electromyographic detection

**Fig. 2:**
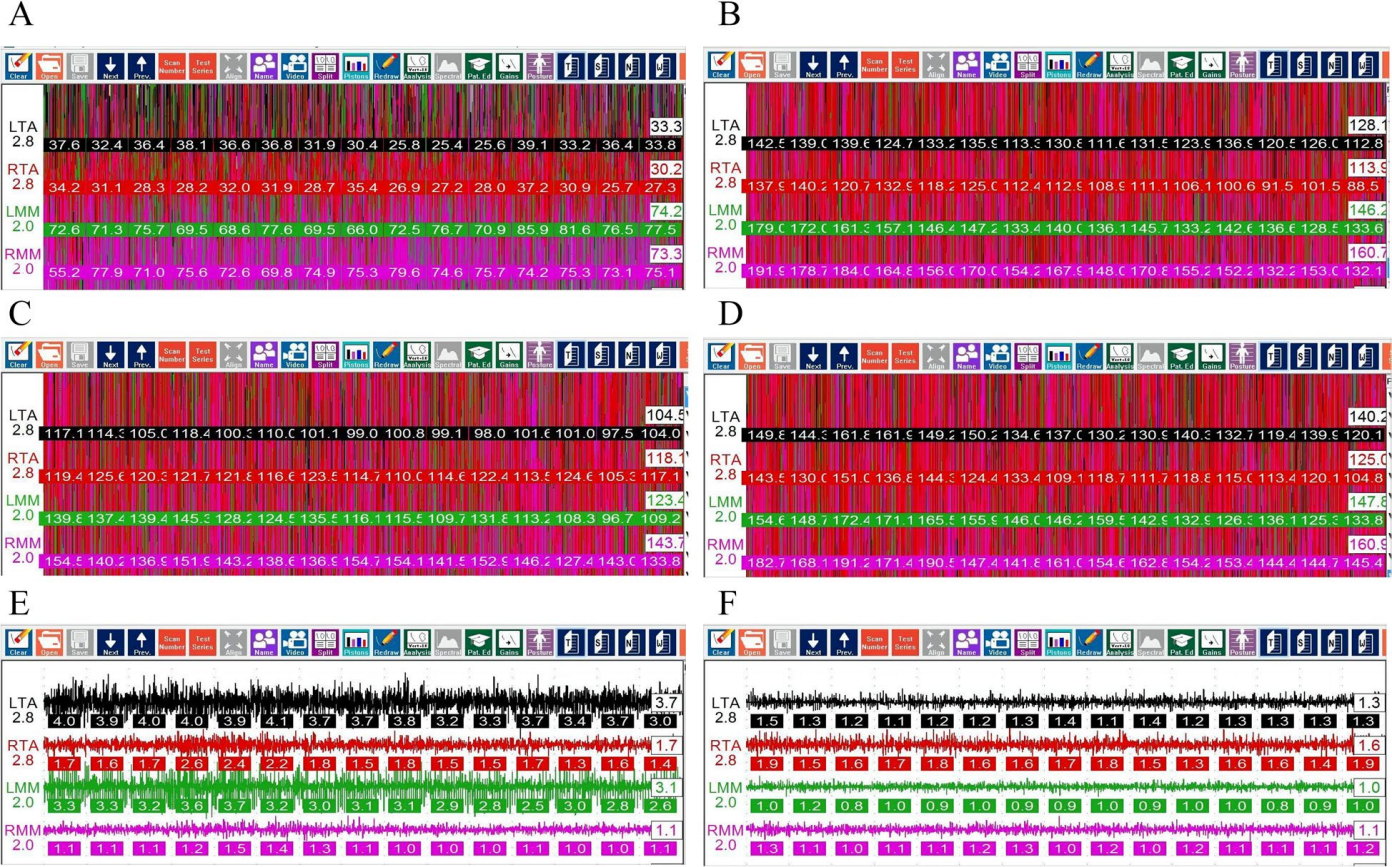
Electromyographic detection at teeth clenched position and rest position. **A** Electromyographic detection at teeth clenched position with use of modified anterior splint. **B** Electromyographic detection at teeth clenched position with use of soft occlusal splint. **C** Electromyographic detection at teeth clenched position without splint. **D** Electromyographic detection at teeth clenched position with use of hard occlusal splint. **E** Electromyographic detection at teeth rest position before J5 TENS. **F** Electromyographic detection at teeth rest position after J5 TENS

Electromyographic activity from masseter muscle at teeth clenched position were compared in participants with three different splints and without splint. EMG data were significantly lower in the participants with modified anterior splint than with hard, soft occlusal splint or without splint (*p* < 0.05). There was no significant difference in participants with use of hard occlusal splint, soft occlusal splint and without splint in terms of EMG values for masseter (*p* > 0.05) (Fig. 2) (Table 1).

**Table 1:** Electromyography records of anterior temporal and masseter muscles with use of different occlusal splints or without splint at teeth clenched position

**Masticatory muscle**		**Without splints** **1** (**Mean ± SD**)	**Soft occlusal splint** **2** (**Mean ± SD**)	**Hard occlusal splint** **3** (**Mean ± SD**)	**Modified anterior splint** **4** (**Mean ± SD**)	**P**						
						1/2/3/4	1/2	1/3	1/4	2/3	2/4	3/4
**Anterior temporal muscle**	**left**	148.63 ± 45.87	154.56 ± 37.52	149.96 ± 39.43	52.03 ± 16.85	< 0.05	> 0.05	> 0.05	< 0.05	> 0.05	< 0.05	< 0.05
	**right**	146.38 ± 45.85	156.97 ± 46.61	157.36 ± 49	59.15 ± 22.48	< 0.05	> 0.05	> 0.05	< 0.05	> 0.05	< 0.05	< 0.05
**Masseter**	**left**	165.73 ± 61.34	168.64 ± 55.19	169.63 ± 67.97	87.30 ± 36.53	< 0.05	> 0.05	> 0.05	< 0.05	> 0.05	< 0.05	< 0.05
	**right**	163.11 ± 63.75	168.07 ± 55.46	170.32 ± 71.84	91.17 ± 38.86	< 0.05	> 0.05	> 0.05	< 0.05	> 0.05	< 0.05	< 0.05

The EMG values of anterior temporal muscle in subjects with hard occlusal splint, soft occlusal splint, and without occlusal splint were significantly higher than with use of the modified anterior splint at teeth clenched position (*p* < 0.05). There was no statistically significant difference in the EMG data for the participants with use of hard occlusal splint, soft occlusal splint and without splint (*p* > 0.05) (Fig. 2) (Table 1).

### Occlusal force evaluation with Dental Prescale II (DP2)

**Fig. 3:**
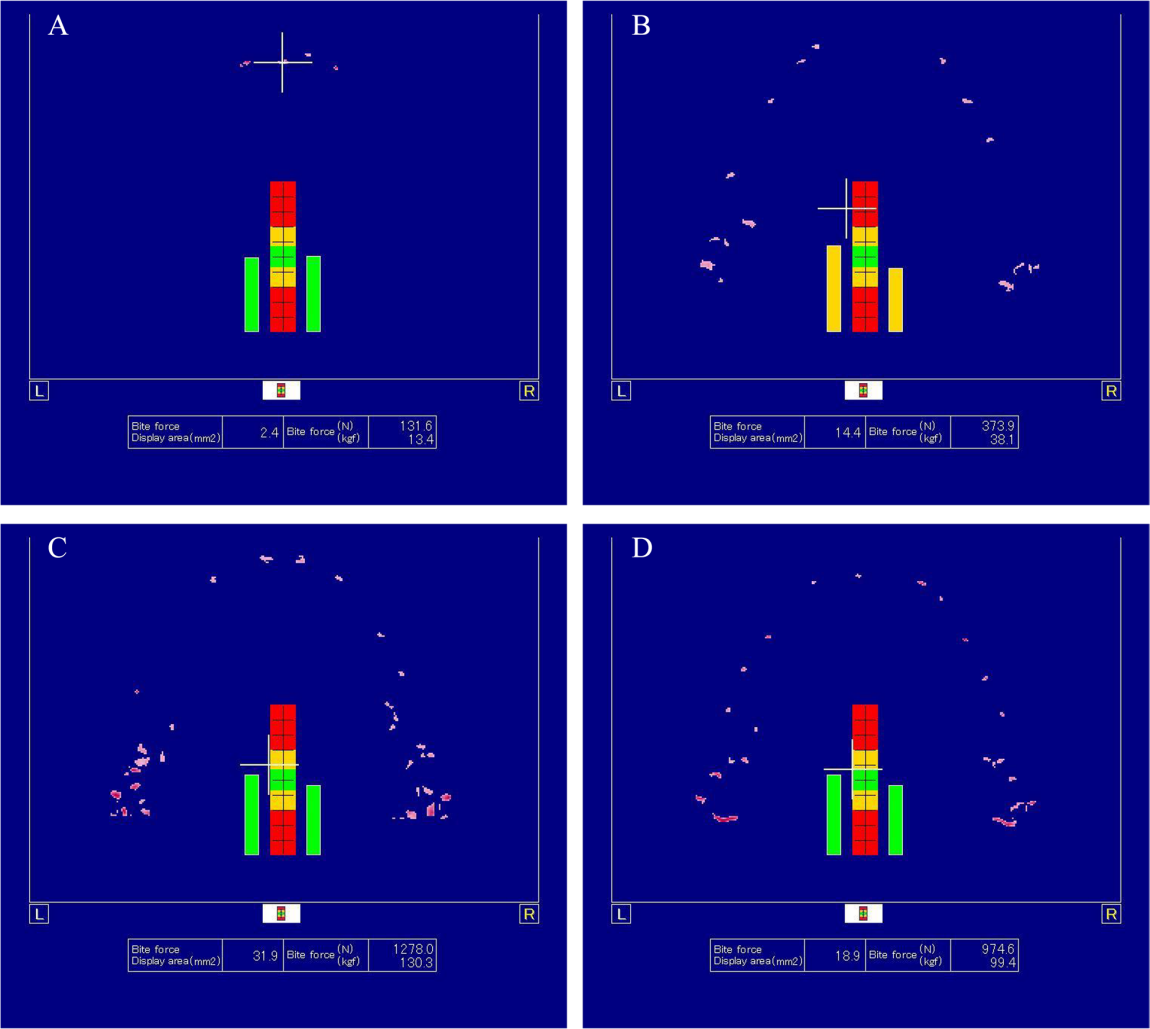
Occlusal force evaluation at teeth clenched position. **A** Occlusal force evaluation at teeth clenched position with use of modified anterior splint. **B** Occlusal force evaluation at teeth clenched position with use of soft occlusal splint. **C** Occlusal force evaluation at teeth clenched position without splint. **D** Occlusal force evaluation at teeth clenched position with use of hard occlusal splint

From the presented data, it can be seen that the maximum bite force and bite area occurred in subjects without use of splint , while the minimal bite force and bite area occurred in subjects with use of modified anterior splint. Bite force and bite area in subjects with use of hard occlusal splint were greater compared with use of soft occlusal splint (Table 2) (Fig. 3).

**Table 2:** Bite force and bite area records of participants with use of different occlusal splints or without splint at teeth clenched position

**Occlusion**	**Without splints** (**Mean ± SD**)	**Soft occlusal splint** (**Mean ± SD**)	**Hard occlusal splint** (**Mean ± SD**)	**Modified anterior splint** (**Mean ± SD**)	**P**						
					1/2/3/4	1/2	1/3	1/4	2/3	2/4	3/4
**Bite force**	1023.07 ± 271.61	259.32 ± 113.29	703.45 ± 257.86	140.94 ± 57.75	< 0.05	< 0.05	< 0.05	< 0.05	< 0.05	< 0.05	< 0.05
**Bite area**	25.84 ± 6.59	9.64 ± 3.89	17.59 ± 6.69	2.63 ± 1.21	< 0.05	< 0.05	< 0.05	< 0.05	< 0.05	< 0.05	< 0.05

### Intermaxillary space and electromyographic activity at rest position pre/post-treatment by J5 TENS

The result of intermaxillary space and EMG level of anterior temporal muscle and masseter pre/post-treatment by J5 TENS were presented in Table 3, which showed the effect of J5 TENS on participants were: 1) muscle activity of the anterior temporalis and masseter reduced during mandible at resting posture after J5 TENS; 2) intermaxillary space increased during mandible at resting posture after J5 TENS.

**Table 3:** Intermaxillary space and electromyographic activity at rest position pre/post-treatment by J5 TENS

**J5 TENS**	**Intermaxillary space** (**Mean ± SD**)	**Anterior temporal muscle** (**Mean ± SD**)		**Masseter** (**Mean ± SD**)	
		**Left**	**Right**	**Left**	**Right**
**before J5**	2.18 ± 0.61	3.08 ± 0.78	2.95 ± 0.85	2.46 ± 0.99	2.36 ± 1.12
**after J5**	3.82 ± 0.86	1.04 ± 044	1.18 ± 043	1.09 ± 0.38	1.14 ± 0.22
**P**	< 0.05	< 0.05	< 0.05	< 0.05	< 0.05

## Discussion

Bruxism is very harmful, but the cause is not clear, so the current treatment methods are diverse [24, 25], including psychotherapy, pharmacological therapy [26–28], biofeedback [29, 30], occlusal repositioning, etc. Occlusal repositioning includes irreversible occlusal therapy (orthodontic therapy, dental prosthesis, occlusal adjustment) and reversible occlusal therapy (intraoral occlusal splint therapy). Among them, occlusal splint application is recommended as the first choice for bruxism treatment because of its low trauma, effective relieving orofacial symptoms and reduction of the complications occurrence [31–34].

There are different types of splints. Accoding to the range of the dentition covered, it can be divided into the part and full coverage occlusal splint. A full coverage occlusal splint with complete tooth contacts can help to avoid direct contact between upper and lower teeth or implant, acts as a stress relaxer to ameliorate the extra excessive overload generated due to bruxism, create a physiological function adaptive change, form a new biomechanical equilibrium between the generated occlusal forces and the physiological loading [35], reduce the tooth wear. Keskinruzgar et al. found hard occlusal splint treatments significantly decreased muscle pain and increased mouth opening in individuals with sleep bruxism [36]. Sriharsha et al. reported the soft occlusal splint chould decrease cortisol levels to reduce the stress levels of individuals with sleep bruxism [37], and reduce pain caused by nocturnal bruxism on muscle and TMJ [38]. Akat et al. assessed the full coverage hard occlusal splint and soft occlusal splint on masseter and temporal muscle activity, thickness, and length in patients with bruxism by ultrasonographic and electromyographic. They found EMG values of masseter and temporal muscle in group with hard occlusal or soft occlusal were significantly lower after treatment than before treatment. (*p* < 0.05). In the masseter, there was a significant length changes occurred after hard occlusal splint treatment [39]. Abe et al. research demonstrated the occlusal splint can induce the reduction in rhythmic masticatory muscle activity related to sleep bruxism and the comfort of the occlusal splint may influence sleep quality in individuals with sleep bruxism [40]. In our study, more patients preferred soft splint and modified anterior splint to hard splint in terms of comfort.

The stomatognathic system is mainly composed of the teeth, occlusion, masticatory muscles, and temporomandibular joint. A harmonious and stable relationship under the control of peripheral and central nervous system contributes to stomatognathic system health, once the interrelationship is disrupted, periodontal disease, temporomandibular joint disorder, and masticatory muscle pain, limitation of jaw movement may develop. Occlusion refers to the contact between the teeth, while occlusion force is defined as the forces exercised by the masticatory muscle to teeth. In clinical practices, masticatory muscles function and occlusal force are important parameters for evaluating the stomatognathic system state objectively [41, 42].

K7-J5 neuromuscular analysis system (Myotronics-Noromed, Inc., Tukwila, WA, USA) mainly consists of two parts: K7 EMG electromyography recorder and J5 Myomonitor transcutaneous electrical nerve stimulation (TENS). K7 can automatically detect bilateral masseter and temporal muscle, and calculate the mean value of the electromyographic intensity in 15 s. Precise measurement of bilateral masseter and temporal muscle at rest and other functional states. In our study,compared to with hard occlusal splint, soft occlusal splint or without splint, the reduction of masseter and temporal muscle activity were observed in records with tooth clenched as a result of subjects with modified anterior splint. J5 Myomonitor TENS is the latest product in the Myo-monitor family to promote muscle relaxation and relieve spasm. It can provide two stimulation channels, Channel A mainly acts on the fifth and seventh pairs of cranial nerves to promote the relaxation of facial muscles, while channel B mainly acts on the shoulder muscles to restore the head to a normal postural position. Repetitive depolarized masticatory muscles and shoulder muscles, direct muscles to perform rhythmical contractions, promote blood circulation and metabolism. It is recommended that 40 min is the ideal time for healthy subjects and patients with masticatory system disorders to obtain sufficient muscle relaxation [43].

Once excessive occlusal pressure are imposed on teeth, tactile discrimination of a tooth derived from periodontal mechanoreceptors enable the self-protection mode and induce negative feedback on the activity of the muscles. However, periodontal ligaments of posterior tooth were compressed by prolonged excursive tooth contact during functional or parafunctional mandibular movements, afferent mechanoreceptors would leads to excess contractions in masticatory muscles. Therefore, prolonged occlusal surface engagement adds on excessive muscle contractions to the baseline contractions, which result in clinical appearance of muscular hyperactivity and symptoms of mandibular dysfunction [44]. In this study, results indicated that J5 TENS treatment could effectively reduce the electromyographic activity of masseter and temporal muscle in subjects with bruxism, and increase the intermaxillary space at mandible rest position.

Various materials and methods have been used to determine the location of occlusal contacts and the size of occlusal force for years. Conventional bite registration technology for analyzing occlusion in clinic primarily includes the use of articulating papers, occlusion wax,and shim-stock foil,which are unable to quantify occlusal contacts. Articulating paper and shim-stock foil can only provide the occlude contact by the mark in paper, and provide relatively bite force and contact area by the depth of the color and mark. Correction of occlude contact relies on dentist's interpretation of paper marks and patients occlusal feel feedback. Saliva, moisture in the oral cavity, material and thickness of the paper will influence the accuracy of marks. Therefore, above methods lacks objective accuracy, reliability, and reproducibility [45]. In order to overcome these limitations and provide more accurate and reliable occlusal relationship and occlusal force, digital quantifiable occlusal indicators have been developed, which can be used to objectively present relative or real quantitative occlusal forces. The common quantitative occlusal indicators used in clinic include Piezoelectric transducer (T-scan), Pressure sensitive film (Dental Prescale), Strain gauge transducer (Dentoforce2), Piezoresistive transducer (Flexiforce) and Pressure transducer (GM10) [46]. T-scan can provide relative occlusal force by presenting the percentage of the bite force compared to the maximal occlusal force, while the Dental Prescale II can quantify, record occlusal force and bite area, and compare the difference among different situation. In our study, the minimal bite force and bite area occurred in subjects with use of modified anterior splint, compared with three other situations.

## Conclusion

The results of this study present significant differences in occlusal evaluation between subjects without splint and with use of three different types of occlusal splints (hard, soft, and modified anterior splint). Occlusal splint may be beneficial for reducing bite force and area on teeth. Compared to hard and soft splint, modified anterior splint seems to be more comfortable and effective in both reducing occlusion force and surface electromyographic activity of anterior temporalis and masseter for subjects with bruxism. However, further studies are needed to prove the long-term effectiveness of modified anterior splint for treatment of bruxism.

## Data Availability

The data used and analysed during the current study are available from the corresponding author on reasonable request.
